# Diesel engine performance and emissions with fuels derived from waste tyres

**DOI:** 10.1038/s41598-018-19330-0

**Published:** 2018-02-06

**Authors:** Puneet Verma, Ali Zare, Mohammad Jafari, Timothy A. Bodisco, Thomas Rainey, Zoran D. Ristovski, Richard J. Brown

**Affiliations:** 1 Biofuel Engine Research Facility, Queensland University of Technology (QUT), Brisbane, QLD-4000 Australia; 2 International Laboratory of Air Quality and Health, Queensland University of Technology (QUT), Brisbane, QLD-4000 Australia; 3 School of Engineering, Deakin University, VIC, 3216 Australia

## Abstract

The disposal of waste rubber and scrap tyres is a significant issue globally; disposal into stockpiles and landfill poses a serious threat to the environment, in addition to creating ecological problems. Fuel production from tyre waste could form part of the solution to this global issue. Therefore, this paper studies the potential of fuels derived from waste tyres as alternatives to diesel. Production methods and the influence of reactor operating parameters (such as reactor temperature and catalyst type) on oil yield are outlined. These have a major effect on the performance and emission characteristics of diesel engines when using tyre derived fuels. In general, tyre derived fuels increase the brake specific fuel consumption and decrease the brake thermal efficiency. The majority of studies indicate that NOx emissions increase with waste tyre derived fuels; however, a few studies have reported the opposite trend. A similar increasing trend has been observed for CO and CO_2_ emissions. Although most studies reported an increase in HC emission owing to lower cetane number and higher density, some studies have reported reduced HC emissions. It has been found that the higher aromatic content in such fuels can lead to increased particulate matter emissions.

## Introduction

Rapidly depleting petroleum resources and rising public concern related to climate change has prompted much research into alternative energy resources^1^. The conversion of waste to energy presents an opportunity to replace conventional fuels at the same time as reducing the waste burden on the planet^2^. Solid waste disposal is a serious global issue that is leading to economic and environmental complications^3^. In the manufacturing and automotive industries, there has been an increase in the quantity of waste rubber and tyres^4,5^, an issue that needs a sustainable solution. It has been reported that approximately 1.5 billion waste tyres are generated every year, globally^6^.

Disposing of end-of-life tyres is not easy and inevitably gives rise to some collateral pollution^7^. For this reason, the treatment of scrap tyres is being addressed by the relevant governing bodies and private industry^8^. 48.5 million tyre Equivalent Passenger Units (EPUs) contributed to Australia’s waste in 2009–2010 compared to 41.8 million in 2007–08^9^. An EPU is a standardised measure of the quantity of tyres, given that the weight for a new standard passenger vehicle tyre is 9.5 kg, whereas a used tyre is standardised as 8.0 kg.

**Figure 1:**
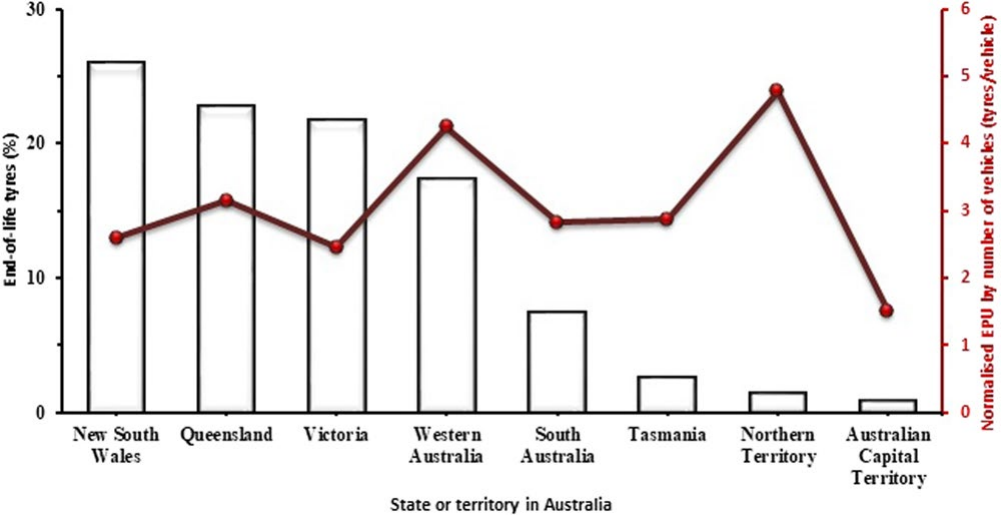
End-of-life EPUs for states or territories in Australia^9^.

The contribution of each Australian state or territory to the total end-of-life tyres (ELTs) in Australia and normalised EPUs by the number of vehicles in 2012 is shown in Fig. 1. New South Wales, Queensland and Victoria dominate the generation of ELTs, which is related to the relatively high number of vehicles in these states^10^. On the other hand, Western Australia and Northern Territory lead in trends for normalised EPU by the number of vehicles in the state, which is most likely due to their low population density and large size. ELTs are converted in tyre recycling plants into tyre crumbs, reinforcing fibre and steel. Styrene butadiene, polybutadiene, nitrile and chloroprene rubbers together with natural rubber are the main constituents of tyre crumbs^5,7–9^. Their chemical structure with aromatic and aliphatic constituents plays an important role in determining the composition of the oils derived from tyre crumbs. The reinforcing fibre is usually recovered as a heterogeneous fluff made up of polymeric fibres that contain rubber^11^.

Tyre pyrolysis oil (TPO), derived from scrap tyres, has been shown to have potential as an alternative to diesel^6,12–19^ with the added benefit of recycling the waste rubber and decreasing our reliance on natural resources^14,20,21^. Pyrolysis involves an oxygen-free thermal decomposition process. The rubber decomposes to form gases and the condensed vapours (i.e. liquids) can be used as fuels^22^. To this end, the recovery of solid and liquid material is achieved^11,23^.

Although there are some literature studies available on the use of waste tyre derived (WTD) fuels, such as TPO, as an alternative fuel to diesel^7,14,24^, these focused on production aspects only. A thorough search of the literature could not find any papers reviewing the engine performance and emission characteristics of TPO in a comprehensive manner. Thus, this paper aims to review the potential of waste TPO to be used as a substitute for diesel and its engine performance, combustion and emission characteristics. This study has been divided into sections as follows: the introduction is given in Section 1, Section 2 follows with a historical overview of the publications related to WTD fuels from waste tyres such as TPO, carbon black and light fraction pyrolysis oil and their application in diesel engines. Section 3 focuses on waste tyres as a source of biofuel, followed by Section 4, which discusses the production techniques. Section 5 contains a detailed analysis of the effect of WTD fuels on engine performance parameters, such as brake specific fuel consumption (BSFC) and brake thermal efficiency (BTE) and exhaust emissions such as nitrogen oxides (NOx), carbon monoxide (CO), carbon dioxide (CO_2_), hydrocarbons (HC), particle mass (PM) and particle number (PN).

## Historical overview

**Figure 2:**
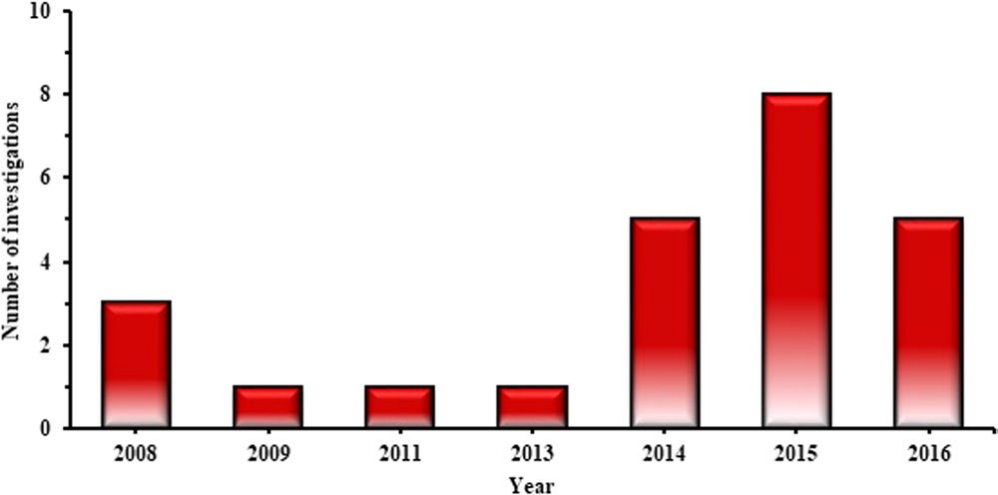
History of published investigations in the use of WTD fuels in a diesel engine (Table A).
- As observed in Fig. 3 (retrieved from Table A), the majority of the studies adopted 5–20% blends of WTD fuel with diesel. However, across the literature the whole spectrum of blend percentages (including pure WTD fuel, 100%) have been represented.

Despite the obvious attraction of producing fuel from waste tyres, there has been a limited amount of literature focused on the use of WTD fuels. Table A, given in the appendix, shows a list of articles published in this field, focussing on the use of WTD fuels in diesel engines. The list of papers given in Table A has been retrieved from Scopus with search terms such as waste tyre pyrolysis oil AND (diesel engine or performance or exhaust or diesel particulate matter); tyre derived fuel AND (diesel engine or performance or exhaust or diesel particulate matter); synthetic fuel AND (diesel engine or performance or exhaust or diesel particulate matter). Some of the papers from the search terms focused on production aspects of WTD fuel, which have been reviewed separately in Section 4. Following the literature listed in Table A, the following has been observed:

- The diesel engines used in the literature have been either direct injection or had a common rail fuel injection system.
- Of the various WTD fuels derived from scrap tyres, the majority of researchers experimented with TPO. However, some research has also focused on fuels from by-products and derivatives of pyrolysis oils such as carbon black, light fraction pyrolysis oil and low sulphur tyre fuel.
- Interest in the use of WTD fuels in diesel engines surged in the late 2000s, as shown in Fig. 2 (retrieved from Table A), and has gained significant attention again recently.

**Figure 3:**
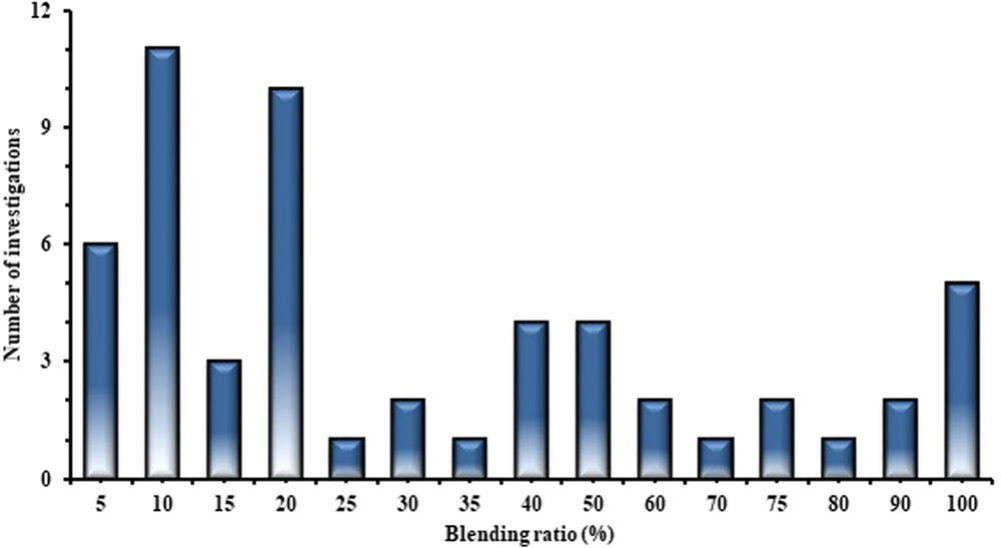
History of published investigations for use of WTD fuels in a diesel engine based on the blending ratio (Table A).
- The majority of the studies analysed diesel engine performance under steady-state conditions. Only three studies investigated transient conditions.
- There is very limited literature reporting the effect of WTD fuels on PM and PN emissions.

## Waste tyre – a source of alternative fuel

Waste tyres pose an environmental issue as they do not readily biodegrade and recovering their constituent components is quite difficult^20^, an issue compounded by the vast quantity of waste tyres discarded annually^6^. Tyres are primarily made of rubber (45–65 wt. %), carbon black (21.5–35 wt. %), and steel (16.5–25 wt. %), but also consist of zinc, sulphur and additives^25^. In addition, the composition varies according the application of the type e.g. a car tyre typically has 14% natural rubber and 27% synthetic rubber whereas truck tyres normally have more natural rubber (27%) and lesser synthetic rubber (14%)^26^. The rest of the components such as fillers, chemical additives (e.g. sulphur), plasticisers and metals are the same for car and truck tyres^26^. The rubber component is present as hydrocarbons compounded with fibrous materials^7^.

Most of the research related to waste tyres has focused on the production of fuel (TPO) which has been used for blending with diesel. In addition, researchers have also attempted to blend carbon black with diesel^27^. Carbon black is the solid waste collected in the pyrolysis reactor upon completion of the pyrolysis of waste tyres^28^. It has been found that ELTs from passenger cars have more sulphur and aromatic content compared to ELTs from heavy duty trucks^29^. Table 1 gives the breakdown of tyre recycling and recovery processes in Australia.

**Table 1:** Summary of domestic tyre recycling and recovery markets^29^.

Form	Destination	Typical applications	Size	Proportion in market (%)
Whole tyres/shredded tyres	Tyre derived fuel	Energy recovery (e.g. pyrolysis)	>200 mm	8.7
Whole tyres	Stockpiling for future recycling	Pyrolysis, crumbing	Not applicable	31
Whole tyres/granules/shredded tyres	Civil engineering	Civil construction	10–60 mm	11
Granulated, crumbed or powdered	Rubber crumb, granules, landfill	Road construction, explosives, adhesives, disposal and flooring	1–10 mm	49.3

As seen in Table 1, the major portion of recycled rubber (49.3%) is granulated or powdered and disposed of in landfills. Crumb rubber can be used to replace the traditional polymer modified binder in spray seal pavements for its plasticity and waterproofing properties. Recycled rubber granulate can be used in a range of moulded products, flooring and matting, which can be used in sporting grounds and playgrounds. In 2013–2014, only 8.7% of the waste tyres were used to produce fuel^29^. Thus, there is excellent potential for producing biofuel from ELTs via the pyrolysis process. As a value-add this will help reduce the financial burden on government bodies to treat waste rubber and prevent landfilling^29^.

## Production methodologies and fuel characterisation

**Figure 4:**
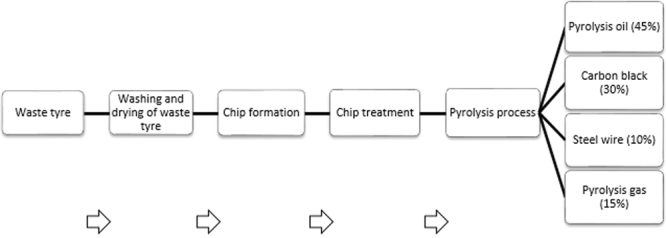
Process chart for different steps in waste tyre pyrolysis^16,28,32,44,75^.

Different studies have been conducted to convert waste automobile tyres into liquid fuels^30–32^. Figure 4 shows the typical process steps in the pyrolysis of waste tyre rubber.

Initially during vehicle tyre treatment, tyres are cut into smaller sized pieces followed by the removal of fabrics, beads and steel wires. This leads to chip formation, which includes chipping thick portions of rubber from the periphery of the tyre. During chip treatment, small rubber chips are washed, dried and fed into a pyrolysis reactor. In the final step, products from pyrolysis in vapour form are fed into a water-condenser and ultimately stored in the form of a liquid fuel. Upon distillation, TPO is collected which is then analysed for its fuel character.

**Figure 5:**
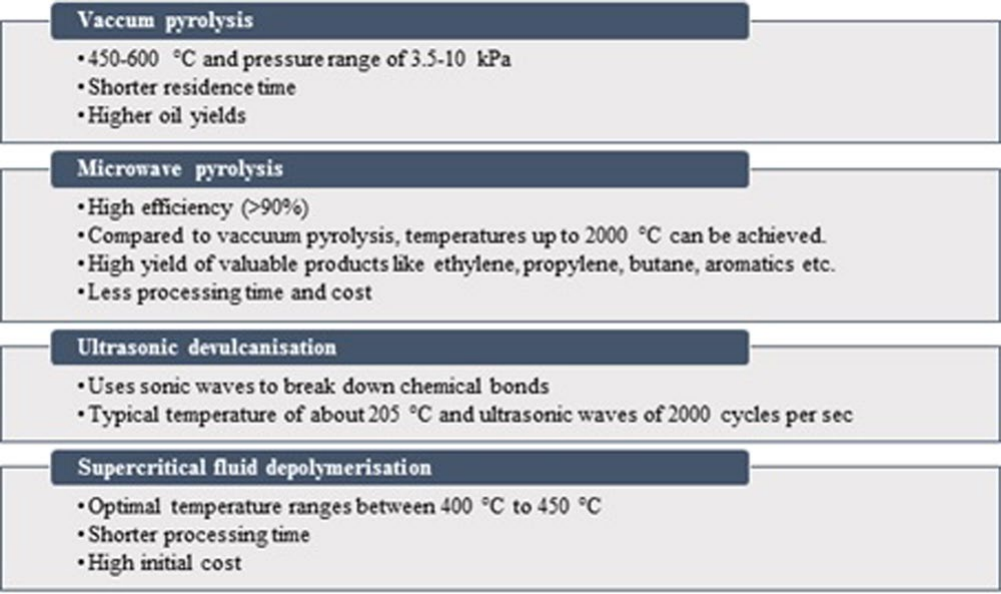
Different technologies for pyrolysis of waste tyres^12,22,39,76^.

Pyrolysis involves the thermal breakdown of biomass at high temperature. The initial temperature for pyrolysis is approximately 400 °C and reactions can continue to occur up to 1000 °C^26^. Much research has focused on the conversion of waste tyres into fuel using a broad range of pyrolysis processes^4,6,11,22,33–36^. Pyrolysis can be classified in terms of several approaches: according to residence time (e.g. fast pyrolysis, intermediate and slow pyrolysis)^37^, reactor type (e.g. fixed, moving, fluidised bed and vacuum reactor pyrolysis) or novel technologies (e.g. microwave and ultrasonic)^12,38^. Fast pyrolysis offers some promising advantages in the conversion of biomass. Generally, fast pyrolysis (residence time < 2 s) is employed to maximise the liquid product yield, while slow pyrolysis is employed to maximise the solid product yield. Pyrolysis has shown potential for transforming used tyres into three components of useful products: carbonaceous solids, liquid hydrocarbons and non-condensable gases^32^. Some major pyrolysis variants such as vacuum pyrolysis^39^, and microwave pyrolysis^3^. Ultrasonic devulcanisation^38,40^ and supercritical fluid depolymerisation^41,42^ are shown in Fig. 5. It has been mentioned in the literature that compared to vacuum pyrolysis, rubber tyres are found to be poor microwave absorbers^3,8,43^. In order to make the process more efficient, several materials such as particulate carbon, glycerol, biomass char, ionic liquids and graphite have been used which aid in improving the microwave absorbing capacity of rubber tyres^8^.

### Effect of reactor operating parameters

The following description of the effect of reactor operating parameters, such as temperature and catalyst (type and concentration), is provided to give a brief overview. Note, this is provided as supplementary information to lay the foundation for exploring the performance (BSFC and BTE) and emission characteristics (NOx, CO, CO_2_, HC and PM emissions) of a diesel engine fuelled with WTD fuels. For interested readers, a detailed review of reactor parameters for pyrolysis process can be found elsewhere^26^.

#### Effect of temperature

In general, researchers have investigated the effect of reactor temperature ranging between 200–700 °C. A reaction temperature over 500 °C has been found to have a less noteworthy effect on fuel yield and composition^4^. Products obtained were found to be a complex mixture of C_5_-C_20_ organic compounds with a high fraction of aromatic compounds and high calorific value (42 MJ/kg)^4^. This was further verified by Murillo *et al*.^23^ who varied the reaction temperature from 400 to 600 °C and observed that 500 °C was the optimum temperature. A similar variation in temperature from 400–700 °C at 50 °C increments was investigated by İlkılıç and Aydın^17^ and it was observed that as the temperature reached 400 °C, the first signs of the liquid product were observed. Liquid yield increased with increase in temperature and achieved its peak value at 500 °C and then dropped marginally to become stagnant. Higher temperatures had a negligible influence on liquid yield. On other hand, gaseous yield kept on increasing at an approximately linearly rate with an increase in reactor temperature. Similar results were observed in another study by the same authors^35^.

There have been other studies in the literature which focused on the effect of reactor temperature during pyrolysis within the range of 200–600 °C^30,44^. It has been observed that the char yield dropped significantly with a rise in pyrolysis temperature. The liquid fuel yield increased when the temperature increased to 350 °C from 280 °C. Further increases in temperature resulted in a drop of the liquid fuel yield. On the other hand, gas yield kept on increasing with increases in temperature from 280–400 °C^30^. In addition to the effect of the reactor temperature, different zeolite catalysts, ultra-stable Y-type (USY) and Zeolite Socony Mobil–5 (ZSM-5) have also been studied^44^. It was observed that for the USY catalyst, 400 °C was the optimal temperature, beyond this temperature the oil yield decreased and the gas yield increased. Similar behaviour was found for the ZSM-5 catalyst. Conesa *et al*.^45^ observed that higher liquid oil yields were obtained at a medium temperature range (300–500 °C) and that for the higher temperature range (>500 °C) the oil yield was found to drop and a higher gas yield acquired.

Some studies have reported the effect of varying other reactor parameters, such as heating rate and flow rate, along with the effect of temperature. Banar *et al*.^46^ experimented with two different heating rates viz. 5 °C/min and 35 °C/min between 350 and 600 °C reactor temperature and observed that an increase in the heating rate resulted in a decrease in the oil yield. The maximum oil yield dropped to 31.1% at a heating rate of 35 °C/min compared to that of 38.8% at 5 °C/min. It was noted that for both heating rates the oil yield increased up to 400 °C and then the commonly reported downward trend observed. Frigo *et al*.^47^ produced diesel like fuel from the pyrolysis of scrap tyres and varied the temperature from 300 to 500 °C. The authors mentioned that the yield of different products was reliant on the flow rate of crushed tyres into a reactor. They achieved a maximum oil yield of ~45% by optimising the flow rate of the crushed tyres.

Temperature effects were investigated in the microwave assisted pyrolysis of scrap tyres with and without activated carbon as a catalyst^48^. For pyrolysis without a catalyst, an increase in temperature resulted in a decrease of char yield initially which then reversed—the opposite trend was observed for the gas yield. The optimum temperature for microwave pyrolysis without a catalyst was found to be between 550–600 °C, the oil yield achieved was between 19.03 to 28.63% (in contrast, with use of a catalyst the maximum oil yield (54.39%) was achieved at 500 °C).

From the above studies, it can be concluded that: the temperature affects the product yields from the pyrolysis reaction significantly; an increase in pyrolysis temperature causes a decrease in yield of carbon black, whereas the yield of gaseous products always increases with temperature; and, the liquid product yield initially increases with an increase in temperature then drops with further increases in temperature.

#### Effect of catalyst

Catalyst type and concentration play an important role in pyrolytic treatment of waste tyres. Shah *et al*.^30^ varied the catalyst (calcium carbide) to tyre rubber ratio from 0.1 to 0.5 and observed that the maximum oil conversion occurred at a ratio of 0.3. Furthermore, it was observed that using a 0.3 calcium carbide to crushed tyre ratio increased the liquid fuel conversion to 38.4%, from 22.8% when no catalyst was used. Boxiong *et al*.^44^ also reported that the use of catalysts USY and ZSM-5 increased the conversion percentage for oil yield in the pyrolysis reaction. The oil yield increased to 70.8% for USY and 56.8% for ZSM-5 compared to that of 35% at non-catalytic conditions.

Dung *et al*.^49^ experimented with two different catalysts at 500 °C during slow pyrolysis: Mobil Composition of Matter No. 41 (MCM-41) prepared with silatrane; and Ru/MCM-41. Compared to the uncatalysed reaction, the oil yield increased with MCM-41 and further increased with Ru/MCM-41. On the other hand, the gas yield decreased and char yield did not change significantly. The presence of acidic sites promotes the conversion of heavy compounds to lighter ones owing to cracking activity, thereby increasing the gas yield. From the above studies, it is seen that the use of different catalysts such as USY, ASM-5, MCM-41 and activated carbon has been beneficial for increasing product yield in waste tyre pyrolysis.

### Fuel characterisation

Fuel properties play a key role in engine performance and emission characteristics. Table 2 shows the comparison of fuel properties of different WTD fuels. Apart from TPO, studies have investigated the carbon black and also TPO which has been distilled. The density of diesel is lower than that of other fuels. The higher oxygen content found in TPO decreases the calorific value, which in turn reduces engine power. In addition, the higher kinematic viscosity of TPO is related to the degree of unsaturation, and it also increases with the oxygen content of the fuel. This can adversely affect the atomisation of the fuel spray and evaporation characteristics of the fuel during combustion. Sulphur and aromatic content is substantially higher in TPO compared to diesel.

**Table 2:** Fuel properties for different WTD fuels^28,32,52,77^.

Fuel property	Diesel	TPO	Distilled TPO	Carbon black
Density @ 15 °C (kg/m^3^)	0.8–0.83	0.92–0.935	0.871	0.86
Flash point (°C)	50	43	36	—
Kinematic viscosity at 40 °C (cSt)	2	3.2	1.7	—
Calorific value (MJ/kg)	42.7–43	38–42.8	45.6	32.6–32.86
Oxygen (% m/m)	—	0.10–3.96	—	1
Sulphur (% m/m)	<0.001	0.72–0.96	0.03	0.02
Carbon (% m/m)	87	83.45–85.60	—	86.4
Nitrogen (% m/m)	—	0.40–1.05	—	0.3
Hydrogen (% m/m)	13	9.59–11.73	—	2.86
Ash content (%)	0.01	0.31	—	10.24
Aromatic content (% m/m)	26	39.3–63	—	—

## Engine performance and emissions

There are a number of studies in the literature which have focused on engine operation with waste TPO blended with diesel. Engine operation analysis of TPO is divided into two sections, performance characteristics and emission characteristics.

### Performance characteristics

Some authors have attributed reduced engine performance (BTE) on TPO fuel to its lower calorific value. Wang *et al*.^6^ observed that BSFC increased with TPO percentage in blended fuels. This was attributed to the lower calorific value of TPO fuel (42.21 MJ/kg) compared to diesel (43.8 MJ/kg). Lower calorific value directly effects BSFC as more fuel is consumed to operate the engine for the same power output. Ilkiliç and Aydin^17^ also concluded that the lower calorific value of TPO contributed to decreased engine power and higher BSFC for diesel-TPO blends. For higher blends of TPO, engine power and torque decreased gradually. Engine power was lowest for pure TPO compared to blends of diesel with 75% and 50% TPO, the reason cited to which was lower calorific value of TPO. BSFC for TPO fuel for 5 to 100% blend increased from 322.91 to 367.17 g/kWh, which was again related to the lower calorific value of TPO.

**Figure 6:**
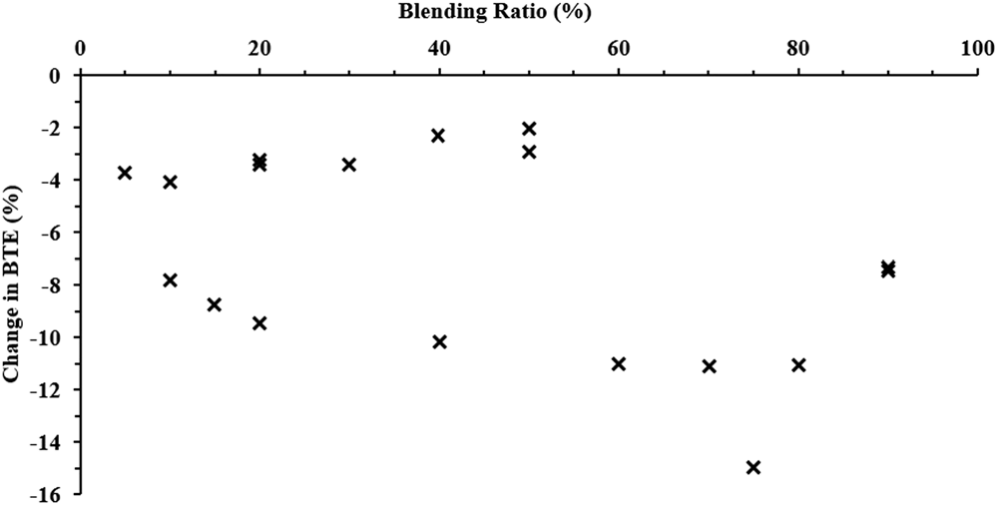
Variation in BTE for different blending ratios of biodiesel compared to neat diesel^15,16,32,51,52,60^.

**Figure 7:**
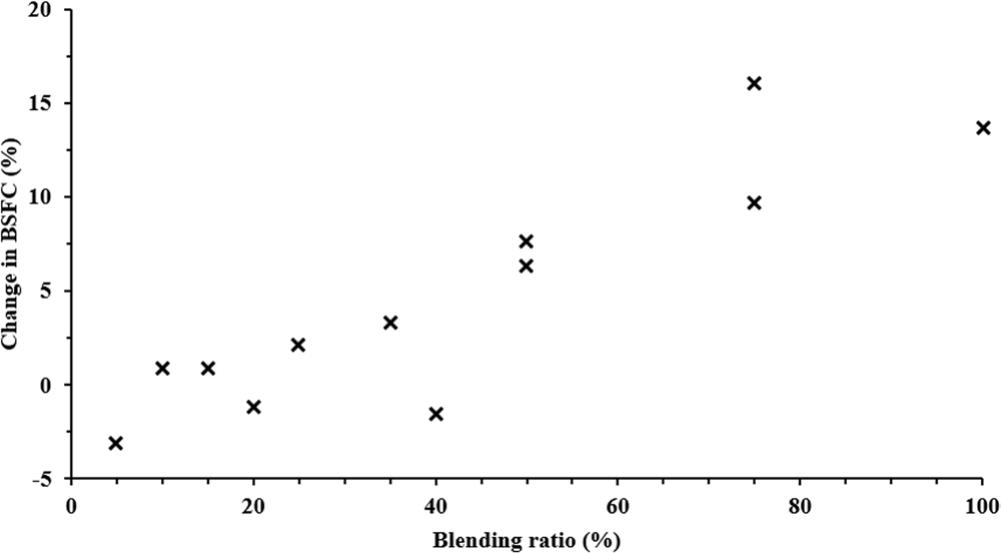
Variation in BSFC for different blending ratios of biodiesel compared to neat diesel^17,60,65^.

Figures 6 and 7 show the variation in BTE and BSFC for different blending ratios of WTD fuels, as retrieved from Table A. It has been observed that BTE decreases with an increase in blend percentage, whereas BSFC increases for higher blends of WTD fuels. Martinez *et al*.^50^ compared the characteristics of TPO and diesel fuel and observed that even a 5% addition of TPO caused an increase in BSFC at low engine loads. A 4% increase in BSFC of the TPO blended fuel was accounted for based on the lower energy content of TPO5 i.e. 42.23 MJ/kg in comparison to 42.31 MJ/kg of neat diesel fuel. At high engine loads, BSFC and BTE were found to be comparable for both types of fuels. High density of TPO fuel is helpful in getting comparable performance characteristics at high engine loads. Fuel composition (cold filter plugging point, viscous fraction and residue after distillation) plays a key role in deciding the BTE and BSFC at low loads because of poor atomisation of fuel and encounters difficulties in mixing with air at low temperatures in the combustion chamber. On the other hand, at higher engine loads the difference in BTE and BSFC for diesel and a blend of 5% of TPO is reduced because the ratio of mechanical to pumping losses-to-fuel energy is reduced for higher loads. Hariharan *et al*.^18^ also reported that the lower BTE of the TPO blended fuel is due to the lower heating value compared to diesel. They also reported that the lower heat release rate (HRR) during the premixed combustion phase is the reason for the lower thermal efficiency of TPO fuel operation up to part load.

Some studies relate the lower BTE and higher BSFC of TPO blended fuel to density and viscosity. Tudu *et al*.^15^ reported that the higher density of TPO fuel (910 kg/m^3^) compared to diesel (830 kg/m^3^) results in poor atomisation and spray characteristics, causing incomplete combustion of fuel. Wamankar and Murugan^51^ also concluded that the higher density and viscosity of carbon black led to poor atomisation, thus, had higher BSFC compared to diesel. Murugan *et al*.^12^ used 10% to 50% of TPO blended in diesel and observed that TPO blended fuels had lower BTE compared to diesel, which was due to higher viscosity and lower heating value of TPO fuel. However, BTE for TPO blends improved with an increase in TPO percentage, though remained lower than that of diesel. Improvement in BTE, BSFC and brake specific energy consumption (BSEC) for higher blends of TPO can be attributed to better lubricity of the blend due to the additional use of TPO. Tudu *et al*.^19^ also said that the presence of aromatic content and higher boiling point of light fraction pyrolysis oil are the key reasons behind higher useful work for TPO blended fuel compared to neat diesel.

In another study, Murugan^36,52^ studied the effect of distillation on TPO and used this fuel in a diesel engine. It was observed that 30% blends of TPO and distilled TPO had marginally lower BTE compared to neat diesel. The authors related the reduced BTE for blends with distilled TPO to a decrease in viscosity. In their study, the fuel spray did not propagate as deeply into the combustion chamber and therefore some fuel remained unburnt. This incomplete combustion resulted in a reduction in efficiency. Further, a 30% blend of distilled TPO with diesel had lower BTE than a 30% blend of TPO with diesel, which might have been due to the presence of volatile fractions resulting in further incomplete combustion.

Wamankar and Murugan^53^ studied the influence of different injection timings and nozzle opening pressures on a direct injection diesel engine operated with a slurry of carbon black, water and diesel. Maximum BTE was observed at an advanced injection timing of 26° before top dead centre (BTDC) and 220 bar nozzle opening pressure. Advancement in injection timing increased the in-cylinder pressure. The maximum peak pressure also occurred at an earlier crank angle because combustion started relatively earlier. On the other hand, an increase in nozzle pressure resulted in a shorter ignition delay. The authors stated that advanced injection timings resulted in a higher heat release rate, thus, reflecting the higher BTE.

### Emission characteristics

Different researchers have studied emission characteristics i.e. NOx, CO, HC, CO_2_, PM and PN emissions of diesel engine operated on TPO, diesel and biodiesel and results are summarised below.

#### Nitrogen oxide emissions

**Figure 8:**
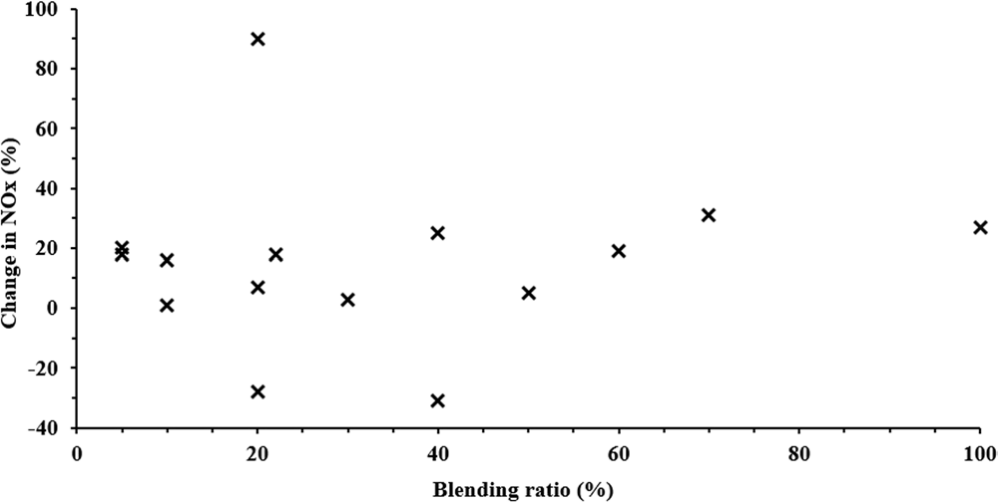
Variation in NOx for different blending ratios of biodiesel compared to diesel^16,32,50,52,57,65,77^.

The fundamental mechanisms behind NOx formation in diesel engines are thermal, prompt, and fuel-bound nitrogen. One of the sources of nitrogen for NOx formation during combustion of diesel and alternative fuels is atmospheric (molecular) nitrogen. Most of the literature studies showed varying trends in terms of the influence of alternative fuels on NOx emission from diesel engines^54^. Thermal NOx refers to the NOx formed through the high temperature oxidation of nitrogen (N_2_) in the combustion chamber. NO emission formation in diesel engine is driven by the following key parameters i.e. in-cylinder temperature, air/fuel ratio, oxygen concentration and residence time for the reaction to take place^51^. In general, different authors have reported an increase in NOx emission with the use of TPO compared to diesel, as observed from the trend given in Fig. 8. Barring a few studies, with an increase in blend percentage, NOx emissions were found to increase with TPO compared to diesel.

Broadly, NOx variation can be discussed on the basis of two parameters: injection related and temperature related.

1. Injection related parameters:

- The use of fuels with higher bulk modulus in hydraulically operated injectors (not in common rail systems) leads to an earlier pressure rise and advances the start of injection. Advanced injection-timing results in an increase in ignition delay, residence time, and duration of premixed phase, thus, resulting in higher in-cylinder temperature and increased NOx formation. Additionally, higher oxygen content allows fuel to premix completely during ignition delay resulting in more heat release during the premixed-burn phase of combustion at ignition and thus accountable for an increase in NOx emissions^55^. Wamankar and Murugan^53^ stated that advancing the injection timing results in higher NO emissions for all fuels owing to rapid combustion and high in-cylinder temperature. Kegl^56^ stated that injection timing is more important than degree of in-cylinder temperature and HRR. Advanced injection results in the maximum in-cylinder temperature and HRR being reached earlier in the cycle, which greatly affects NOx emissions.
- Differences in the viscosity of fuels also plays a key role in advanced fuel injection systems operating with a mechanical pump. Fuels with low viscosity have increased fuel leakage, leading to a decrease in the rate of pressure rise and thus delays in the start of injection^54^. In general TPO has higher viscosity than diesel^16,57^, therefore less fuel leakage and higher pressure rise, which ultimately results in advanced injection timing.

2) Temperature related parameters:

- Higher nitrogen content in pure TPO fuel (0.79 wt.%) compared to diesel (0.00 wt.%)^50,58^ is a further reason behind the noted increase in nitrogen oxides.
- Additionally, higher adiabatic flame temperatures increase NOx formation. More aromatic content, lower H/C ratio and fuel bound oxygen result in higher adiabatic flame temperature for engine operation with TPO fuel^50,58^, which results in higher NOx emissions^59^.

Contrary to the general trend in the published literature, some studies reported a decrease in NOx emissions with the use of TPO fuel.

- Murugan *et al*.^52^ related NOx variation to the stoichiometry of fuel and in-cylinder temperature. It has been reported that when the in-cylinder temperature goes beyond 1800 K, NOx formation starts^60^. If there is lean stoichiometry during the combustion process, NOx formation is lower, which is generally at lower load conditions. On other hand, due to the diffusive mixing of fuel and air occurring along the spray envelope, combustion takes place near stoichiometric, forming higher NOx. It was reported that lower blends of distilled TPO (10% to 20%) with diesel have low NOx emissions, compared to higher blends such as 90% blend of distilled TPO resulting in an increase in NOx, which was linked to higher HRR in the case of higher blends.
- Aydın and İlkılıç^60^ related lower NOx emission for low sulphur TPO with in-cylinder temperature. It was concluded that NOx emissions reduced by 29.3 and 40.2% for 50% and 75% blends of low sulphur TPO compared to diesel. The primary reason cited for lower NOx emission for low sulphur TPO blended fuel was lower in-cylinder temperature.
- Other important factors that lead to a reduction in NOx are considered to be the lower cetane number of low sulphur tyre fuel, which results in delayed combustion and higher heat of vaporisation that results in decreased combustion temperature^60^. Tudu *et al*.^19^ stated that lower peak pressure and in-cylinder temperature are the key reasons for lower NO emissions for fuel blended with light fraction pyrolysis oil (LFPO) compared to neat diesel.
- Further, it has been reported that operating the engine with exhaust gas recirculation (EGR), aids in inhibiting NOx formation. Martinez *et al*.^50,58^ used TPO blended fuel in a diesel engine and expressed that higher EGR values lowers the oxygen concentration and flame temperature, thereby helping in reducing thermal NOx emissions.

#### CO and CO_2_ emissions

**Figure 9:**
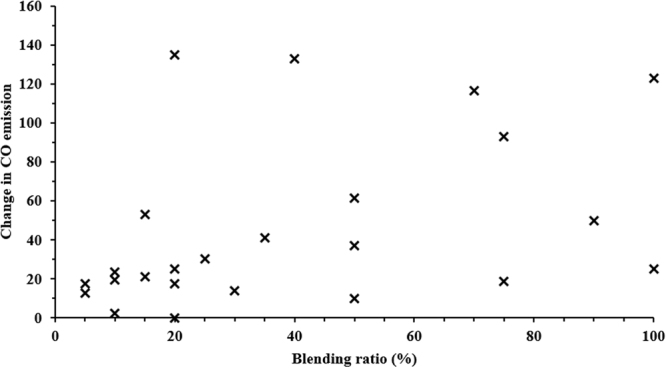
Variation in CO emissions for different blending ratios of biodiesel compared to diesel^16,17,28,32,52,60,65,77^.

CO emissions are the result of incomplete combustion typically due to a lack of oxygen or available time in the cycle for the completion of combustion. In general, an increase in engine load or speed results in a decrease in CO emissions; however, an excessive increase in engine speed results in higher CO emissions. At very high engine speeds, a lower combustion flame temperature limits CO oxidation into CO_2_; therefore, the CO emission values at very high engine speeds tend to also be higher^53,60,61^. Literature relating to the use of TPO in diesel engines indicate an increase in CO emissions with TPO blended fuel^17,19,52,60^, which is clear from the trend given in Fig. 9. With an increase in blending ratio, CO emissions gradually increased compared to diesel, although attempts have been made to decrease the CO emissions with use of TPO by blending it with other fuels such as biodiesel^15,57^.

Ilkiliç and Aydin^17,60^ stated that higher density of TPO fuel results in more injection of fuel on a mass basis leading to lower air-to-fuel ratio, which has been considered crucial for higher CO emissions due to decreased availability of air. This was further supported by Wamankar *et al*.^51^ and Tudu *et al*.^19^ higher fuel density leads to lower air-to-fuel ratio. Moreover, the higher viscosity of TPO results in poor atomisation characteristics of fuel resulting in incomplete combustion, further compounding the issue with CO emissions. Contrary to general statements in the literature, Murugan *et al*.^52^ stressed a leaner fuel air mixture in the cylinder and said that the flame does not propagate through some of the mixtures nearer to the wall and crevice volume. This causes incomplete combustion leading to higher CO emission for distilled TPO blended fuel when compared to diesel.

There have been attempts made to reduce the CO emissions even with use of WTD fuels. Koc and Abdullah^57^ observed that all the different biofuel blends (TPO or biodiesel) had CO emissions lower than neat diesel. At lower engine speeds, a 10% blend of biodiesel with diesel had the highest CO emission, whereas, 10% each of biodiesel and TPO in diesel (80% diesel) had the lowest CO emissions. Tudu *et al*.^15^ concluded that using 10% dimethyl carbonate (DMC) in light fraction pyrolysis oil resulted in a 66% reduction in CO emissions. This could be due to the additional oxygen content of DMC, resulting in a more homogeneous mixture of air-fuel resulting in more complete combustion. The other reasons may be the turbulent motion by an internal jet helps make the air-fuel mixture inside the combustion chamber more homogeneous, which leads to more complete combustion^15^. With an advancement in fuel injection timing, 16–18% lower CO emissions are observed, which is mainly due to higher cylinder temperatures and more rapid oxidation between C and O_2_ molecules. Retardation in fuel injection timing causes CO emissions to increase^53^.

Similar to CO emissions, lower engine speeds under load result in higher CO_2_ emissions^57^. Completely oxidised carbons in the fuel give rise to higher CO_2_ emissions. Ternary blends like 5% to 10% each of biodiesel and TPO in diesel had lower CO_2_ emission than neat diesel^57^. Tudu *et al*.^15^ found an improvement in CO_2_ emission for fuel blended with LFPO or DMC compared to neat diesel. Additionally, provision of turbulence with help of internal jet piston arrangement helps in further reduction of CO_2_ emission^15^.

#### Hydrocarbon emissions

The unburnt hydrocarbon emissions are a direct result of incomplete combustion, due to the incomplete mixing of the air and fuel. The concentration of unburned hydrocarbon decreases with engine load. HC emissions for different emulsions were found to be higher than diesel due to the higher density of emulsion fuels, resulting in poor atomisation^51^.

**Figure 10:**
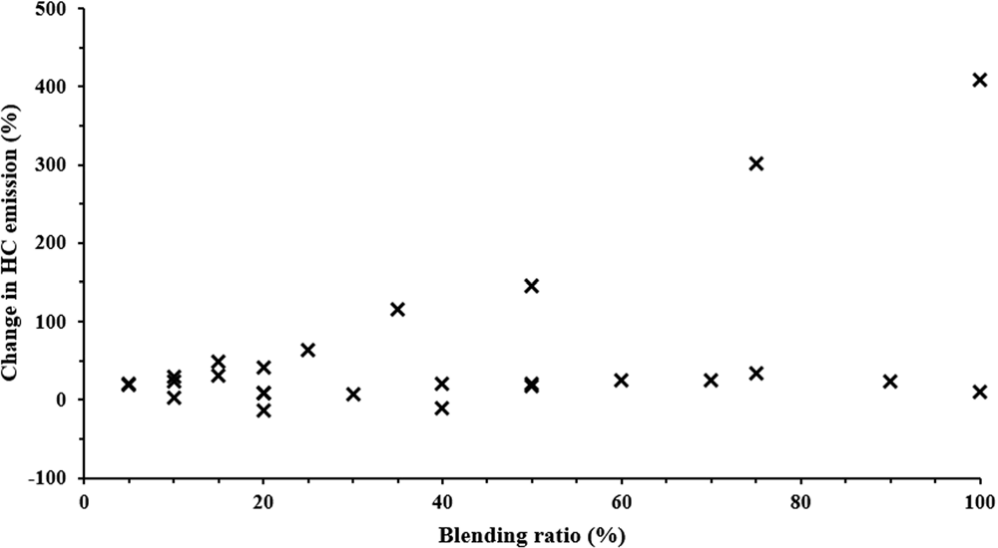
Variation in HC emissions for different blending ratio of biodiesel compared to diesel^16,17,28,32,52,60,65,77^.

In general, the literature reports an increase in HC emissions for WTD fuels, as observed in the trend (Fig. 10). Murugan *et al*.^52^ stated that an increase in HC emissions for distilled TPO blended fuel was due to the presence of unsaturated hydrocarbons, which did not break down during the combustion process. The literature also reports that the fuel spray does not propagate into the combustion chamber properly, resulting in gaseous hydrocarbons on the inner walls of the cylinders and crevice volumes being left unburnt, causing incomplete combustion.

Aydın and İlkılıç^17,60^ related the lower cetane number of low sulphur TPO blended fuel, which resulted in higher ignition delay. In addition, higher density and final distillation temperature in low sulphur TPO blends were the main reasons behind the higher HC emissions, compared to diesel. Murugan *et al*.^32^ said that poor volatility, higher density and viscosity are the key reasons for higher HC emission with TPO blended fuel. Similar observations were made by Wamankar and Murugan^28,51,53,62–64^ for the emulsion of carbon black, water and diesel. In this study, higher HC emissions were related to poor atomisation of the fuel, as a consequence of: the higher density and viscosity, inferior fuel spray quality and a lower compression ratio of the fuel. Tudu *et al*.^15^ also reported higher HC emissions for a fuel blended with the light fraction of pyrolysis oil, but observed a reduction with the use of an internal jet piston. This difference was attributed to higher turbulence by the internal jet piston motion. Martinez *et al*.^58^ related higher HC emission for TPO fuel due to higher sulphur content compared to diesel fuel.

In contrast to the general finding, some authors reported a decrease in HC emissions for TPO blended fuels. Frigo *et al*.^65^ reported that the relatively lower viscosity (2.9 cSt) of TPO compared to diesel (3.5 cSt) resulting in better spray atomisation and that the aliphatic hydrocarbon and aromatic content aided TPO vaporisation and ultimately the combustion velocity. All of these factors contributed towards lower HC emissions compared to diesel. Öztop *et al*.^66^ observed lower HC emissions for TPO blended fuel compared to gasoline at higher engine speeds; however, they observed an increase in HC emission at low engine speed. The reason for this could be poor atomisation at low engine speeds due to the higher fuel viscosity. However, at higher engine loads, air movement causes a more favourable air-to-fuel ratio, resulting in complete combustion, thereby reducing HC emission.

#### Diesel particulate matter emissions

PM is a solid and liquid mixture suspended in a gas. In combustion chambers of compression ignition engines, fuel is injected and mixed with an oxidant; consequently, a great degree of heterogeneity characterises the combustion process. This process, which is the main cause of PM emission, is called diffusion flame combustion^67^. PM is a complicated pollutant that is not chemically well-defined in terms of its composition, formation and control. PM composition depends on factors such as, engine speed and load, fuel type, lubricant type, engine maintenance, after-treatment systems and the level of dilution after being emitted^68,69^.

TPO has the potential of reducing PM emissions owing to its oxygen content, which ranges from 0.10–3.96 wt%. (as shown in Table 2). Most studies in the literature have indicated that alternative fuels such as biodiesels can decrease PM emissions^59,70,71^ owing to their oxygen content^70–72^. Soot formation generally occurs in the fuel-rich zone, under high temperature decomposition during combustion. It mainly takes place in the fuel spray core region. Since these fuels are partially oxygenated, the fuel-rich zone is reduced, preventing higher soot formation and aiding the soot oxidation process leading to lower PM emissions^59^. However, PM formation is affected by different parameters which can either reinforce or cancel the effect of one another under different conditions.

Recently, PN emissions, which is a count of individual particles, has gained more attention. This could be due to the toxicity of particles, which increases by decreasing the particle size^73^. The EU Commission included PN in the Euro 5b and Euro 6 emission standards for light-duty and heavy-duty vehicles^74^. However, there is still a lack of regulation on the size of emitted particles. Small particles could be more harmful to health because they can penetrate deeper into the lungs and cause inflammation where deposited^73^.

Martinez *et al*.^58^ found that a 5% blend of TPO with diesel exhibited higher smoke and PN emissions compared to diesel. This could be due to the higher aromatic and sulphur content. Lower biodiesel blends show comparatively lower smoke emissions because of better combustion and fuel atomisation^28^. Martinez *et al*.^50^ concluded that the higher aromatic content in TPO fuel is a key factor in increasing PM emissions because it aids the formation of soot precursors, resulting in higher sooting tendency. In addition, authors could not find any research in the literature studying the morphology and nanostructural characteristics of PM emissions from diesel engine fuelled with WTD fuels.

## Conclusion

The use of alternative fuels in diesel engines has been attracting researchers over the last decade owing to the gradual decrease in petroleum-based fuels and their subsequent environmental concerns. There has been 48.5 million tyre EPUs of waste in Australia alone in 2009–2010 and the rate of waste accumulation has been increasing. Using waste tyre pyrolysis oil could be a solution to this issue owing to the treatment of waste rubber and scrap tyres by recycling. There have been various studies on pyrolysis of scrap tyres, production of WTD fuels and their use in diesel engines. This review paper studied the historical overview of the literature published on the production of WTD fuels from waste tyres and their application in diesel engines. The application of WTD fuels in diesel engines started mainly in the late 2000s and has gained the attention of researchers as alternative fuels to diesel. Most of the studies have focused on the use of 5 to 20% of WTD fuel blended in diesel, whereas, some studies have even tried complete replacement of diesel. This paper reviewed the production aspects of WTD fuels from waste tyres such as methodologies, effect of operating parameters e.g. reactor temperature and catalyst. Further, fuel properties, engine performance and emission characteristics for WTD fuels were reviewed with the following findings

1. Among different pyrolysis processes, microwave and vacuum pyrolysis stand out due to the high yield of products and relatively lower processing time.
2. Reactor temperature plays a key role in the pyrolysis process. It was observed that an increase in temperature increases the oil yield initially, but after achieving the peak, oil yield drops gradually. On the other hand, the gas yield increases and char yield decreases with an increase in reactor temperature. The optimum temperature for the pyrolysis process has been found to be within 450 °C–500 °C.
3. The type of catalyst and its concentration also holds an important role in the pyrolysis process. It has been concluded that the catalyst to rubber ratio within 0.3–1% to be optimum and USY, ASM-5, MCM-41 catalysts have been found to be most effective.
4. TPO has a higher oxygen content (0.10–3.96%) compared to diesel, which could be effective in the reduction of PM emissions.
5. For performance parameters, in general BSFC and BTE are found to be higher for use in TPO blended fuel compared to diesel possibly due to a lower caloric value (38–42.8 MJ/kg). In addition, higher density and viscosity were also cited as the reasons for poor atomisation of the fuel leading to inferior BSFC and BTE.
6. For emission characteristics, mixed observations have been reported for NOx emissions. Some authors reported an increase in NOx emissions with use of TPO blended fuel, attributed mainly to higher viscosity and the higher nitrogen and oxygen content of TPO fuel. On other hand, some report a decrease in NOx emissions, which was attributed to lower peak pressure and in-cylinder temperature and lower cetane number.
7. CO and CO_2_ emissions have been found to be higher with the use of TPO blended fuel due to the higher density and viscosity of TPO, leading to a lower fuel to air ratio. A similar was the trend observed for HC emissions.
8. There has not been significant literature on DPM with TPO. Although, due to the higher oxygen content, PM emissions should be lower than that for diesel but it has been reported that the higher aromatic content in TPO resulted in higher PM and PN emissions none-the-less. There is a significant gap and scope of research to analyse PM emission for TPO blended fuel in the future.

## Electronic supplementary material

Supplementary Information
