# Supplementary material for: Diesel engine performance and emissions with fuels derived from waste tyres
Source: Sci Rep. 2018 Feb 6;8:2457. doi: 10.1038/s41598-018-19330-0 (PMC5802751; doi:10.1038/s41598-018-19330-0)
Supplement: Supplementary file 1 — Supplementary Information [file 41598_2018_19330_MOESM1_ESM.pdf]

Table A: Details of literature reviewed focusing on use of WTD fuels in diesel engine

Research group	Reference	Year	Publication	Fuel	Engine type	Biodiesel blending ratio (%)	Operation mode	Performance parameters	Emission characteristics
Murugan et al.	[1]	2008	Fuel	TPO	Four stroke, air cooled, single cylinder with rated power of 4.4 kW at 1500 rpm.	20, 90	Steady-state	BSFC, BTE, Exhaust gas temperature	NOx, CO, HC, Smoke
Murugan et al.	[2]	2008	Waste management	TPO	661cc, single cylinder air-cooled direct injection diesel engine, rate power 4.4 kW at 1500 rpm.	10, 30, 50	Steady-state	BTE, Exhaust gas temperature	NOx, HC, CO, Smoke
Murugan et al.	[3]	2008	Fuel Processing Technology	Distilled TPO	661cc, single cylinder air-cooled direct injection diesel engine, rate power 4.4 kW at 1500 rpm.	80, 90	Steady-state	BTE, BSEC, Exhaust gas temperature	NO, HC, CO, Smoke
Murugan et al.	[4]	2009	Fuel Processing Technology	TPO	Single cylinder, four stroke, CI, water cooled, rated power 3.7 kW at 1500 rpm	20, 40, 60, 70	Steady-state	BTE, BSEC	NO, HC, CO, Smoke
Ilkiliç and Aydin	[5]	2011	Fuel Processing Technology	TPO	DI engine, 406 cc, 10 HP at 3600 rpm, Air cooled	5, 10, 15, 25, 35, 50, 75, 100	Steady-state	Power, Torque, EGT, BSFC	CO, NOx, HC, SO_2, Smoke
Hariharn et al.	[6]	2013	Fuel	TPO	661cc, single cylinder air-cooled direct injection diesel engine, rate power 4.4 kW at 1500 rpm.	100	Steady-state	BTE, BSEC	NOx, HC, CO, Smoke
Wamankar and Murugan	[7]	2014	Fuel Processing Technology	Carbon black	661cc, single cylinder air-cooled direct injection diesel engine, rate power 4.4 kW at 1500 rpm.	5, 10, 15, 20	Steady-state	BSEC, Exhaust gas temperature	CO, HC, NO, Smoke
Frigo et al.	[8]	2014	Fuel	TPO	442 cc, DI engine, air cooled, rated power 7.7 kW at 3600 rpm	20, 40	Steady-state	Brake power, BSFC	HC, CO, NOx
Martinez et al.	[9]	2014	Fuel	TPO	4-cylinder, turbocharged, intercooled, 2.0 L diesel engine	5	Transient	BSFC, BTE	CO, NOx, HC, PM

Research group	Reference	Year	Publication	Fuel	Engine type	Biodiesel blending ratio (%)	Operation mode	Performance parameters	Emission characteristics
Koc and Abdullah	[10]	2014	Fuel Processing Technology	TPO with biodiesel	3.3 L, light duty diesel engine, 4-cyinder, rated power 60 kW at 2200 rpm, water cooled 13.	5, 10	Steady-state	Torque, Power, BSFC, EGT	NOx, CO, CO2
Martínez et al.	[11]	2014	Applied Energy	TPO	4-c14.ylinder, turbocharged, intercooled, 2.0 L diesel engine, light duty	5	Transient	BSFC	THC, NOx, CO, Smoke, PN, PM
Sharma and Murugan	[12]	2015	Energy Conversion and Management	TPO with biodiesel	661cc, single cylinder air-cooled direct injection diesel engine, rate power 4.4 kW at 1500 rpm.	20	Steady-state	BTE, BSFC, BSEC	CO, HC, NO, Smoke
Aydın and İlkılıç	[13]	2015	Fuel	Low sulphur TPO	406cc air cooled, direct injection diesel engine with rated power of 10 HP at 3600 rpm.	50, 75	Steady-state	Torque, BSFC, Exhaust gas temperature	NOx, CO, CO_2, HC, smoke emission, O_2, excess air
Vihar et al.	[14]	2015	Fuel	TPO	Heavy duty, Turbocharged, 6-cylinder, 6870 cc DI engine, rated power 162 kW at 2400 rpm, water cooled	100	Steady-state	Torque, BSFC	NOx, THC, CO, Smoke, SO_2
Wamankar and Murugan	[15]	2015	Energy	Carbon black	661cc, single cylinder air-cooled direct injection diesel engine, rate power 4.4 kW at 1500 rpm.	10	Steady-state	BTE, Exhaust gas temperature	CO, NO, HC, smoke
Wamankar and Murugan	[16]	2015	Energy	Carbon black	661cc, single cylinder air-cooled direct injection diesel engine, rate power 4.4 kW at 1500 rpm.	5, 10, 15, 20	Steady-state	BTE, Exhaust gas temperature	CO, NO, HC, smoke
Wamankar and Murugan	[17]	2015	Journal of the Energy Institute	Carbon black	661cc, single cylinder air-cooled direct injection diesel engine, rate power 4.4 kW at 1500 rpm.	10	Steady-state	BTE, Exhaust gas temperature	CO, NO, HC, smoke
Wamankar and Murugan	[18]	2015	Journal of the Energy Institute	Carbon black	661cc, single cylinder air-cooled direct injection diesel engine, rate power 4.4 kW at 1500 rpm.	10	Steady-state	BTE, BSEC	NOx, HC, CO, Smoke

Research group	Reference	Year	Publication	Fuel	Engine type	Biodiesel blending ratio (%)	Operation mode	Performance parameters	Emission characteristics
Wamankar et al.	[19]	2015	Energy	Carbon black	661cc, single cylinder air-cooled direct injection diesel engine, rate power 4.4 kW at 1500 rpm.	10	Steady-state	BTE, Exhaust gas temperature	CO, HC, NO, Smoke
Tudu et al.	[20]	2016	Fuel	Light fraction pyrolysis oil	661cc, single cylinder air-cooled direct injection diesel engine, rate power 4.4 kW at 1500 rpm.	40	Steady-state	BTE, Exhaust gas temperature	HC, CO, CO_2, NO, Smoke
Tudu et al.	[21]	2016	International Journal Oil, Gas and Coal Technology	Light fraction pyrolysis oil	661cc, single cylinder air-cooled direct injection diesel engine, rate power 4.4 kW at 1500 rpm.	20, 40, 60	Steady-state	BSFC and Brake Power	NO, Smoke, CO
Daniel et al.	[22]	2016	International Journal of Ambient Energy	TPO	Single cylinder, constant speed, water cooled DI engine, natural aspiration, rated power 3.7 kW at 1500 rpm	10, 20, 30, 50	Steady-state	BSFC	CO, HC, NO, Smoke
Pilusa, T.J.	[23]	2016	Waste management	TPO	6-cylinder, turbocharged diesel engine, rated power 206 kW at 2220 rpm	100	Transient	Torque, Power, BSFC	CO, HC, SO_2
Wang et al.	[24]	2016	Applied Thermal Engineering	TPO	2800cc 4-cylinder water-cooled diesel engine.	10, 20	Steady-stare	BSFC, BP	SO_2
Vihar et al.	[25]	2017	Energy Conversion and Management	TPO	1560cc, 4-cylinder water-cooled diesel engine, rated power 66 kW at 4000 rpm.	100	Steady-state	-	NOx, HC, CO, PM
Sharma and Murugan	[26]	2017	Applied Thermal Engineering	TPO with biodiesel	661cc, single cylinder air-cooled direct injection diesel engine, rate power 4.4 kW at 1500 rpm.	20	Steady-state	BSEC	NO, HC, Smoke

References

1. Murugan, S., Ramaswamy, M. C. & Nagarajan, G. A comparative study on the performance, emission and combustion studies of a DI diesel engine using distilled tyre pyrolysis oil-diesel blends. *Fuel* **87,** 2111–2121 (2008).

2. Murugan, S., Ramaswamy, M. C. & Nagarajan, G. The use of tyre pyrolysis oil in diesel engines. *Waste Manag.* **28,** 2743–2749 (2008).

3. Murugan, S., Ramaswamy, M. C. & Nagarajan, G. Performance, emission and combustion studies of a DI diesel engine using Distilled Tyre pyrolysis oil-diesel blends. *Fuel Process. Technol.* **89,** 152–159 (2008).

4. Murugan, S., Ramaswamy, M. C. & Nagarajan, G. Assessment of pyrolysis oil as an energy source for diesel engines. *Fuel Process. Technol.* **90,** 67–74 (2009).

5. Ilkiliç, C. & Aydin, H. Fuel production from waste vehicle tires by catalytic pyrolysis and its application in a diesel engine. *Fuel Process. Technol.* **92,** 1129–1135 (2011).

6. Hariharan, S., Murugan, S. & Nagarajan, G. Effect of diethyl ether on Tyre pyrolysis oil fueled diesel engine. *Fuel* **104,** 109–115 (2013).

7. Wamankar, A. K. & Murugan, S. Experimental investigation of carbon black-water-diesel emulsion in a stationary di diesel engine. *Fuel Process. Technol.* **125,** 258–266 (2014).

8. Frigo, S., Seggiani, M., Puccini, M. & Vitolo, S. Liquid fuel production from waste tyre pyrolysis and its utilisation in a Diesel engine. *Fuel* **116,** 399–408 (2014).

9. Martinez, J. D., Rodriguez-Fernandez, J., Sanchez-Valdepenas, J., Murillo, R. & Garcia, T. Performance and emissions of an automotive diesel engine using a tire pyrolysis liquid blend. *Fuel* **115,** 490–499 (2014).

10. Koc, A. B. & Abdullah, M. Performance of a 4-cylinder diesel engine running on tire oil-biodiesel-diesel blend. *Fuel Process. Technol.* **118,** 264–269 (2014).

11. Martinez, J. D., Ramos, A., Armas, O., Murillo, R. & Garcia, T. Potential for using a tire pyrolysis liquid-diesel fuel blend in a light duty engine under transient operation. *Appl. Energy* **130,** 437–446 (2014).

12. Sharma, A. & Murugan, S. Potential for using a tyre pyrolysis oil-biodiesel blend in a diesel engine at different compression ratios. *Energy Convers. Manag.* **93,** 289–297 (2015).

13. Aydın, H. & İlkılıç, C. Analysis of combustion, performance and emission characteristics of a diesel engine using low sulfur tire fuel. *Fuel* **143,** 373–382 (2015).

14. Vihar, R., Seljak, T., Rodman Opresnik, S. & Katrasnik, T. Combustion characteristics of tire pyrolysis oil in turbo charged compression ignition engine. *Fuel* **150,** 226–235 (2015).

15. Wamankar, A. K. & Murugan, S. Combustion, performance and emission characteristics of a diesel engine with internal jet piston using carbon black- water- diesel emulsion. *Energy* **91,** 1030–1037 (2015).

16. Wamankar, A. K. & Murugan, S. Combustion, performance and emission of a diesel engine fuelled with diesel doped with carbon black. *Energy* **86,** 467–475 (2015).

17. Wamankar, A. K. & Murugan, S. DI diesel engine operated with carbon-black-water-diesel slurry at different injection timing and nozzle opening pressure. *J. Energy Inst.* 1–14 (2015). doi:10.1016/j.joei.2015.04.003

18. Wamankar, A. K. & Murugan, S. Effect of injection timing on a di diesel engine fuelled with a synthetic fuel blend. *J. Energy Inst.* **88,** 406–413 (2015).

19. Wamankar, A. K., Satapathy, A. K. & Murugan, S. Experimental investigation of the effect of compression ratio, injection timing & pressure in a DI (direct injection) diesel engine running on carbon black-water-diesel emulsion. *Energy* **93,** 511–520 (2015).

20. Tudu, K., Murugan, S. & Patel, S. K. Effect of tyre derived oil-diesel blend on the combustion and emissions characteristics in a compression ignition engine with internal jet piston geometry. *Fuel* **184,** 89–99 (2016).

21. Tudu, K., Murugan, S. . & Patel, S. K. Experimental analysis of a DI diesel engine fuelled with light fraction of pyrolysis oil. *Int. J. Oil, Gas Coal Technol.* **11,** 318–338 (2016).

22. Daniel, M. P., Kumar, K. V. & Prasad, B. D. Performance and emission characteristics of diesel engine operated on tyre pyrolysis oil with exhaust gas recirculation. *Int. J. Ambient Energy* **37,** 609–615 (2016).

23. Pilusa, T. J. The use of modified tyre derived fuel for compression ignition engines. *Waste Manag.* (2016). doi:10.1016/j.wasman.2016.06.020

24. Wang, W. C., Bai, C. J., Lin, C. T. & Prakash, S. Alternative fuel produced from thermal pyrolysis of waste tires and its use in a di diesel engine. *Appl. Therm. Eng.* **93,** 330–338 (2016).

25. Vihar, R., Urban, Z. B., Siljak, T. & Katrasnik, T. Combustion and emission formation phenomena of tire pyrolysis oil in a common rail Diesel engine. *Energy Convers. Manag.* (2017). doi:10.1016/j.enconman.2017.02.005

26. Sharma, A. & Murugan, S. Effect of Blending Waste Tyre Derived Fuel on Oxidation Stability of Biodiesel and Performance and Emission Studies of a Diesel Engine. *Appl. Therm. Eng.* **118,** 365–374 (2017).
